# Using four‐dimensional computed tomography images to optimize the internal target volume when using volume‐modulated arc therapy to treat moving targets

**DOI:** 10.1120/jacmp.v13i6.3850

**Published:** 2012-11-08

**Authors:** Nikolaos Yakoumakis, Brian Winey, Joseph Killoran, Charles Mayo, Thomas Niedermayr, George Panayiotakis, Tania Lingos, Laurence Court

**Affiliations:** ^1^ Department of Radiation Oncology Dana‐Farber/Brigham and Women's Cancer Center Boston MA USA; ^2^ Department of Radiation Oncology Mayo Clinic Rochester MN USA; ^3^ Department of Medical Physics School of Medicine University of Patras Patras Greece; ^4^ Department of Radiation Physics The University of Texas MD Anderson Cancer Center Houston TX USA

**Keywords:** treatment margins, ITV

## Abstract

In this work we used 4D dose calculations, which include the effects of shape deformations, to investigate an alternative approach to creating the ITV. We hypothesized that instead of needing images from all the breathing phases in the 4D CT dataset to create the outer envelope used for treatment planning, it is possible to exclude images from the phases closest to the inhale phase. We used 4D CT images from 10 patients with lung cancer. For each patient, we drew a gross tumor volume on the exhale‐phase image and propagated this to the images from other phases in the 4D CT dataset using commercial image registration software. We created four different ITVs using the N phases closest to the exhale phase (where N=10, 8, 7, 6). For each ITV contour, we created a volume‐modulated arc therapy plan on the exhale‐phase CT and normalized it so that the prescribed dose covered at least 95% of the ITV. Each plan was applied to CT images from each CT phase (phases 1–10), and the calculated doses were then mapped to the exhale phase using deformable registration. The effect of the motion was quantified using the dose to 95% of the target on the exhale phase $\left(D_{95}\right)$ and tumor control probability. For the three‐dimensional and 4D dose calculations of the plan where N=10, differences in the $D_{95}$ value varied from 3% to 14%, with an average difference of 7%. For 9 of the 10 patients, the reduction in $D_{95}$ was less than 5% if eight phases were used to create the ITV. For three of the 10 patients, the reduction in the $D_{95}$ was less than 5% if seven phases were used to create the ITV. We were unsuccessful in creating a general rule that could be used to create the ITV. Some reduction (8/10 phases) was possible for most, but not all, of the patients, and the ITV reduction was small.

PACS number: 87.55.D‐

## I. INTRODUCTION

Four‐dimensional computed tomography (4D CT) imaging techniques can provide the clinician with much needed patient‐specific information on the motion of lung tumors.^(1)^ In many clinics, the internal target volume (ITV) is drawn as the outer envelope of the tumor motion seen in these 4D CT images, although there are many other techniques,^(2)^ and several groups have investigated the use of different margins when treating moving tumors.^3^, ^5^ Evidence exists that including 100% of the motion within the ITV may be conservative. For example, van Herk et al.^(4)^ simulated the effects of breathing on static conformal fields and found that respiratory motion could be accounted for using an inferior margin of 25% of the peak‐to‐peak amplitude and a superior margin of 45% of the peak‐to‐peak amplitude (i.e., a total margin of 70% of the peak‐to‐peak amplitude). Mutaf and Brinkmann^(5)^ simulated the effects of respiratory motion on static conformal fields by summing 10 dose distributions, each offset according to the breathing phase. Their results, which were in broad agreement with those of van Herk et al., showed that respiratory motion could be accounted for using a total margin of 0.72A – 2.5 mm, where A is the peak‐to‐peak amplitude, thus yielding a total margin approximately 70% of the peak‐to‐peak amplitude.

Some difficulty exists, however, in implementing these margin formulae in current clinical practice. For example, different parts of the tumor may have different peak‐to‐peak amplitudes. Also, the previously mentioned studies did not address the effects of changes in the shape of the tumor as it moves, due to respiration. In this work we used 4D dose calculations, which include the effects of shape deformations, to investigate an alternative approach to creating the ITV. We hypothesized that instead of needing images from all the breathing phases in the 4D CT dataset to create the outer envelope used for treatment planning, it is possible to not use the images from the phases closest to the inhale phase. This hypothesis is based on the expectation that for the majority of the respiratory cycle, the tumor position is close to its position in the exhale phase. This approach would create an ITV smaller than that defined by the outer envelope, consistent with the margin‐formulae approaches. If successful, our approach may be easier to implement clinically than margin formulae, and would use the data in a 4D CT image sets without requiring the technological jump to 4D dose calculations.

## II. MATERIALS AND METHODS

### A. Patient selection

We reviewed the 4D CT images of the first 40 lung cancer patients who had received 4D CT imaging as part of their treatment simulation in 2008, and identified the 10 patients who had the largest tumor motion. Table 1 shows the tumor size, location, and extent of motion estimated from the motion of landmarks in the tumor. The Dana‐Farber Cancer Center institutional review board approved the use of these data.

**Table 1:** Motion and position of the patients' tumors. GTV_50 is the gross tumor volume contoured on the exhale (50%) phase of the four‐dimensional computed tomography (4D CT) image set.

*Patient Number*	$GTV\_50(cc^{3})$	*Extent (amplitude) of Motion (mm)*			*Position (lobe)*
		*AP*	*SI*	*LR*	
1	138	0	15	0	Lower right
2	41	5	5	1.1	Middle right
3	37	0	12	3	Lower left
4	81	0	6	0	Upper right
5	38	0	5	0	Lower left
6	429	0	8	0	Upper right
7	88	0	10	0	Lower right
8	64	0	3	0	Upper right
9	86	3	10	0	Upper left
10	93	2	5	5	Middle right

AP=anteroposterior; SI=superoinferior; LR=left‐right plane.

### B. target delineation

Current clinical practice for target delineation at the Dana‐Farber/Brigham & Women's Cancer Center is for the radiation oncologist to draw the ITV as the outer envelope of the motion of the gross tumor volume (GTV) using the full set of 4D CT images (all phases). They do not draw the GTV itself. For our study, a physicist redrew the GTV on the exhale phase (50%) of the 4D CT image set using the clinically drawn target as a guide.

This newly redrawn GTV was identified as GTV_50. The GTV_50 contour was then deformed to the other nine phases of the 4D CT image set using deformable image registration software^(6)^ (MIMVista, MIM Software Inc., Cleveland, OH), yielding a total of 10 GTV contours — one for each phase of the 4D CT. We visually examined all these GTV contours to confirm the accuracy of the deformation.

Multiple ITVs were then created for evaluation by taking the union of the GTVs created using images from specific phases with the final result copied to the exhale‐phase CT images. These ITV contours were identified as ITV_N/10, where N represented the number of phases used to create the ITV. For example, the ITV that represents our current clinical practice, based on all 10 phases of the 4D CT, was identified as ITV_10/10. We also created ITV_8/10, ITV_7/10, and ITV_6/10 by respectively excluding the 2, 3, and 4 GTVs from breathing phases closest to the inhale phase. To accentuate the impact of the ITV contour on the delivered doses, we used no additional clinical target volume or planning target volume (PTV) margins for treatment planning (i.e., the ITV was considered to be equal to the PTV).

### C. Treatment planning

For each patient, we created volume‐modulated arc therapy (VMAT) plans for each of the four ITV targets (ITV_10/10, ITV_8/10, ITV_7/10, and ITV_6/10) using RapidArc (Varian Medical Systems, Palo Alto, CA). The exhale‐phase CT image was used for optimization and three‐dimensional (3D) dose calculation. The Eclipse 8.6 treatment planning system (Varian Medical Systems) was used for all optimizations and dose calculations. Specifically, we used the Anisotropic Analytical Algorithm dose calculation (AAA) (Varian Medical Systems) and had heterogeneity corrections turned on. The RapidArc plans used either one arc, or two coplanar arcs. In each case, the first arc was a full 360°. The second arc started at the 180° gantry position (i.e., posterior), and moved 140°–180° on the ipsilateral side. Details are given in Table 2.

**Table 2:** Details of the RapidArc plans created for each patient.

*Patient Number*	*Number of Arcs*	*Magnitude of the Second Arc (degrees)*	*Hottest Point Dose (% above prescription dose)*
1	1	‐	11.2
2	1	‐	13.1
3	2	160	12.5
4	2	180	9.1
5	2	130	17.1
6	1	‐	11.1
7	2	150	10.9
8	2	150	12.2
9	2	160	7.1
10	2	140	7.6

For each patient, we kept the prescribed dose the same as that used for the actual original treatment (range: 37.5–66.0 Gy). The target coverage constraint was that at least 95% of the target would receive the prescribed dose. The normal tissue constraints were as follows: less than 50% of the total normal lung volume should receive a dose of 5 Gy or higher ($V_{5}<50\%$), less than 30% of the total normal lung volume should receive a dose of 20 Gy of higher ($V_{20}<30\%$), the mean lung dose should be less than 17 Gy, the maximum cord dose was 50 Gy, and the maximum dose allowed within the 7 mm margin of the spinal cord was 54 Gy. After optimization and dose calculation, we compared the test plans with the original plans used for the actual treatments to ensure that our plans were clinically reasonable. All of the plans for each patient were normalized to have the same dose to 95% of the target volume ($D_{95}$).

We then applied the treatment fields to all the other phases in the 4D CT image set, calculating the dose distribution for each phase separately. The dose from each phase was then deformably mapped to the exhale phase of the 4D CT image set using MimVista's VoxAlign Deformation Engine (MIM Software Inc., Cleveland, OH), and we calculated the cumulative dose and dose‐volume histogram (DVH) for the GTV from the treatment. The calculation of the cumulative dose is identified below as a 4D dose calculation. We visually checked the dose distributions after image deformation.

### D. Dose calculation and plan comparisons

Three‐ and four‐dimensional dose calculations were compared using the value of $D_{95}$ for the GTV calculated on the exhale CT image. When determining whether a smaller ITV could be used, we considered the plan based on ITV_10/10 to be the current standard. Other plans were considered acceptable if the reduction in $D_{95}$ from the 4D dose calculation was less than 5% compared with that for the standard plan based on ITV_10/10. Our choice of 5% is arbitrary, and was selected as a starting point for this study. We also calculated the tumor control probability (TCP) using a linear Poisson distribution,^7^, ^9^ using $D_{50}$ and γ of 51.97 Gy and 1.8, respectively, for non‐small cell lung cancer,^(8)^ and normalizing the prescription to 66 Gy.

## III. RESULTS

Table 3 shows the ratio of $D_{95}$ and TCP for the target (GTV_50) when calculated in 4D on all phases (doses were deformed to the exhale scan) to that calculated in 3D on the exhale CT scan for the plan where the target was included completely in the ITV (i.e., ITV_10/10). The average $D_{95}$ ratio (± standard deviation) was 0.93±0.04 , and the range was 0.86–0.96.

**Table 3:** Comparison of four‐dimensional (4D) and three‐dimensional (3D) dose calculations for the gross tumor volume (GTV) on the exhale‐phase computed tomography (CT) image.

*Patient Number*	*Ratio of* $D_{95}$ *for GTV_50 from 4D dose calculation to that from the 3D dose calculation for the plan using ITV_10/10*	*Ratio of TCP for GTV_50 from 4D dose calculation to that from the 3D dose calculation for the plan using ITV_10/10*
1	0.86	0.96
2	0.96	1.00
3	0.95	0.99
4	0.96	0.99
5	0.90	0.96
6	0.90	0.98
7	0.96	1.00
8	0.92	0.98
9	0.93	0.98
10	0.95	0.99

**Figure 1:**
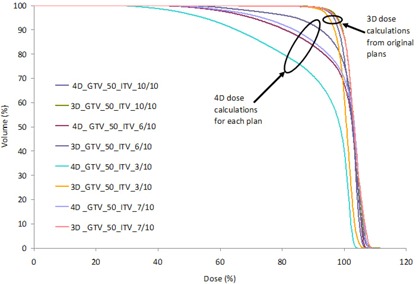
DVHs from the 3D dose calculation (treatment plan) and 4D dose calculations (using all 10 phases of the 4D CT image set) for plans using ITVs created using the union of the GTVs from the N phases closest to the exhale phase, where N=3, 6, 7, and 10. The 3D calculations (on the exhale phase) were all similar. The 4D calculations show the reduction in target coverage as the ITV shrinks

Figure 1 shows the DVH of the GTV_50 from the 3D and 4D dose calculations for patient 1 when different ITVs were used. The DVH of the GTV_50 was very similar for all 3D dose calculations, but the 4D dose calculations showed increasing degradation of the DVH as fewer phases of 4D CT image set were used to create the ITV.

**Figure 2:**
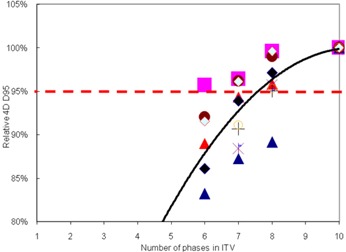
$D_{95}$ value as a function of the number of phases in the 4D CT image set used to calculate the ITV. All values were taken from 4D dose calculations and were normalized to the $D_{95}$ for N=10 (i.e., normalized to current clinical practice). Each marker type is for a different patient. The solid line is the average.

Figure 2 shows how the $D_{95}$ of the GTV_50 for each plan, normalized to the ITV_10/10 plan (4D dose calculations), changed with the number of phases used to create the ITV. When fewer phases were used to create the ITV, the normalized $D_{95}$ decreased. For some patients (e.g., the patient identified with triangles), the decrease in normalized $D_{95}$ was rapid. For others

(e.g., the patient identified with squares) there was little change. The patient with the most rapid decrease (triangles) was the patient with the largest SI motion (patient 1). However, some patients with very little motion (e.g., patient 5) also demonstrated a rapid decrease.

**Figure 3:**
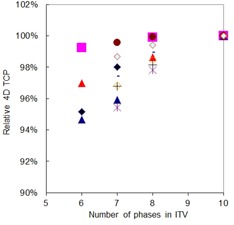
TCP value as a function of the number of phases in the 4D CT image set used to calculate the ITV. All values were taken from 4D dose calculations and were normalized to the TCP for N=10 (i.e., normalized to current clinical practice). Each marker type is for a different patient.

Figure 3 shows how the TCP of the GTV_50 for each plan, normalized to the ITV_10/10 plan (4D dose calculations), changed with the number of phases used to create the ITV. As with the $D_{95}$ data of Fig. 2, when fewer phases were used to create the ITV, the normalized TCP decreased, although the changes in TCP were less than the changes in $D_{95}$.

**Figure 4:**
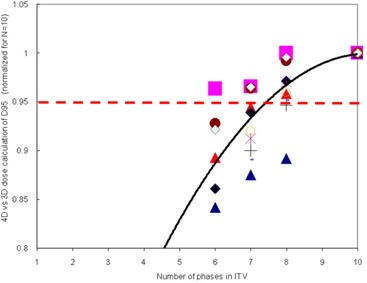
Ratios of the $D_{95}$ values from 4D dose calculations to that calculated in the 3D treatment plan as a function of the number of phases of the 4DCT image set included in calculating the ITV. All data were normalized to the $D_{95}$ for N=10. This very similar to indicating that although there were differences in the treatment plans, these differences did not impact the conclusions of our study. Each marker type is for a different patient. The solid line is the average.

Figure 4 shows the ratio of the 4D to the 3D $D_{95}$ dose calculations for each target, with this ratio normalized to the ratio for the ITV_10/10 plan (i.e., the values in Table 3). As the ITV shrank, the agreement between the 4D and 3D dose $D_{95}$ calculations worsened. This figure attempts to illustrate how variations between treatment plans can affect results. Although there were some differences in the two plots (Figs. 2 and 4), they were very close in shape — indicating that differences in the details of the individual plans should not affect the conclusions of this work.

Table 4 shows for each patient the number of phases that had to be included in the ITV if the $D_{95}$ for GTV_50 from the 4D dose calculations was to remain within 5% of the $D_{95}$ for GTV_50 when all phases were used for the plan. The number of phases that needed to be included ranged from 7 to 10, and there did not appear to be any link between this value and the size or location of the tumor or the extent of its motion. For nine of the patients, it was possible to reduce the number of phases used from 10 to 8, but the associated reductions in ITV were small.

**Table 4:** Details of the RapidArc plans, and the number of phases that can be used to create the internal target volume (ITV) using the criterion that the dose to 95% of the target (D_95_) for the gross tumor volume (GTV) should not be reduced by more than 5% compared with that in the ITV_10/10 plan. Also shown are the possible volume reductions. For all plans, the first arc was 360°. The second arc, if included, started at 180° (i.e., posterior beam) and moved on the ipsilateral side.

*Patient Number*	*GTV_50 (cc)*	*ITV_10/10 (cc)*	*Number of Phases Needed for ITV (5% criterion)*	*ITVopt (cc)*	*ITV Reduction cc (%)*
1	138	190	10	190	0 (0)
2	41	59	6	49	10 (17)
3	37	56	8	48	8 (10)
4	81	105	8	102	3 (3)
5	38	48	8	45	3 (7)
6	429	472	7	461	11 (2)
7	88	129	8	123	6 (5)
8	64	75	8	73	2 (3)
9	86	112	8	106	6 (5)
10	93	118	7	112	6 (5)

## IV. DISCUSSION

The original motivation for this study was published work that it is possible to use ITV margins which are smaller than the magnitude of the motion (as discussed in the Introduction). We, however, found that we could not reduce the ITV margin. Although we found that the ITV could be reduced for some patients, this was not always the case. For 9/10 patients, it was possible to reduce the number of phases included to 8, but the change in target volume, even for these cases, was small (see Table 4). Given that this reduction in the ITV comes with a reduction in dose to the target (we allowed a reduction of 5% for up to 5% of the GTV), there appears to be little gain in attempting to reduce the ITV further. This conclusion is different from those of other researchers (see Introduction) who showed that it was possible to use smaller margins. There are several possible causes for this discrepancy. These include various uncertainties in our work, described below, and also the fact that we included target deformations in the analysis. Further work is necessary to evaluate these differences. This work does, however, support the popular approach of drawing the internal target volume (ITV) as the outer envelope of the tumor motion seen in 4D CT images.

The differences between 3D and 4D dose calculations found in our study were similar in magnitude to those reported by Starkschall et al.^(10)^ They compared 4D and 3D dose calculations for 15 lung cancer patients and found that the 4D dose calculations gave a $D_{95}$ for the PTV of 4.5% ±3.5% (range: from ‐12.3% to +1.7%) lower than that of the 3D dose calculations. Those results are in broad agreement with our findings of a mean decrease of 7% ±4% (range: from ‐14% to ‐4%) for the ITV_10/10 plan.

The main sources of uncertainty in our study were the deformations of the contours used to create the ITVs for the different plans, and the deformations of the dose matrices used to calculate the cumulative dose distributions. Both the contour and dose deformations were checked visually, and no obvious errors were identified. Although the same algorithm has been used in several studies,^6^, ^9^ there has yet to be a fully rigorous evaluation of the accuracy of the registration and dose deformation. A multi‐institutional comparison of different image registration techniques applied to a lung phantom found average errors in predicted marker location of 1.3 to 3.9 mm, depending on the technique, and maximum errors of 5.1 to 15.4 mm.^(11)^ These errors are of the same order of magnitude as the differences in margin size that we investigated in this study. It is possible, therefore, that inaccuracies in deformation may have affected the results presented here, and improved image registration and dose deformation algorithms may give more consistent results. It has also been shown that the errors in 4D dose calculations may be larger in the penumbral region^(12)^ — which is the region which may have largest impact on the results presented here. Of course, this study only evaluated the ITV using the treatment planning (i.e., pretreatment) 4DCT, and variations in respiration and other factors throughout the treatment (inter‐ and intrafraction) would contribute additional uncertainties.

It can be seen in Table 2 that there is a large variation in dose uniformity between the different plans (7%–17%). These differences may affect the optimum margins for each patient. We attempted to reduce the impact of differences in plan design by not only comparing the reductions in coverage as we reduced the ITV, but also the corresponding increases in the difference between 4D and 3D dose calculations. However, these interplan variations will act as an additional source of heterogeneity in the results, making it difficult to give concrete conclusions.

In this study we used 5% as the degree to which $D_{95}$ could be reduced, and the plan still be considered acceptable. This was an arbitrary starting point. It can be argued that this is too loose, and that a 5% reduction is clinically unacceptable. However, even with this 5% level, we were unable to demonstrate noteworthy reductions in ITV. Tightening this level would not change the overall conclusions of this work. We also calculated relative changes in TCP. These changes were smaller than the changes in $D_{95}$. However, reducing the number of phases used to create the ITV from 10 to 8 and 7 gave a reduction in TCP of up to 3% and 5%, respectively.

## V. CONCLUSIONS

In this study, we attempted to appropriately reduce the size of the ITV by reducing the number of phases represented in the 4D CT image set used to draw the outer envelope of the target motion. We evaluated treatment margins for 10 patients and found that although for some patients it may be possible to reduce the size of the ITV, this was not possible for all patients. Even where it was possible to reduce the margins, the actual reductions in ITV were small.
